# Managing RFID Sensors Networks with a General Purpose RFID Middleware

**DOI:** 10.3390/s120607719

**Published:** 2012-06-07

**Authors:** Ismael Abad, Carlos Cerrada, Jose A. Cerrada, Rubén Heradio, Enrique Valero

**Affiliations:** 1 School of Computer Engineering, Universidad de Educación a Distancia (UNED), C/Juan del Rosal, 16. 28040 Madrid, Spain; E-Mails: ccerrada@issi.uned.es (C.C.); jcerrada@issi.uned.es (J.A.C.); rheradio@issi.uned.es (R.H.); evalero@issi.uned.es (E.V.); 2 3D Visual Computing and Robotics Lab, Universidad de Castilla-La Mancha (UCLM), Paseo de la Universidad, 4. 13071 Ciudad Real, Spain

**Keywords:** RFID sensor network (RSN), SCADA (System and Data Acquisition System), device independent acquisition layer, RFID acquisition networks

## Abstract

RFID middleware is anticipated to one of the main research areas in the field of RFID applications in the near future. The Data EPC Acquisition System (DEPCAS) is an original proposal designed by our group to transfer and apply fundamental ideas from System and Data Acquisition (SCADA) systems into the areas of RFID acquisition, processing and distribution systems. In this paper we focus on how to organize and manage generic RFID sensors (edge readers, readers, PLCs, *etc*…) inside the DEPCAS middleware. We denote by RFID Sensors Networks Management (RSNM) this part of DEPCAS, which is built on top of two new concepts introduced and developed in this work: MARC (Minimum Access Reader Command) and RRTL (RFID Reader Topology Language). MARC is an abstraction layer used to hide heterogeneous devices inside a homogeneous acquisition network. RRTL is a language to define RFID Reader networks and to describe the relationship between them (concentrator, peer to peer, master/submaster).

## 1. Introduction

RFID technology has advanced significantly over the past few decades. Rapid advances in microelectronic transceivers have reduced the size and costs of HF and UHF RFID infrastructure, permitting longer reading ranges and faster reading rates than ever before. RFID technology is now able to deal with novel applications with higher mobility and large numbers of tagged objects and software and hardware elements now enable specific functionalities and general services [1] and offer important advantages over other identification mechanisms [2]. However, these new situations require a more robust, flexible and complex middleware platforms in order to resolve issues at different layers of the communication architecture in different application contexts [3]. This complexity has brought up research issues in RFID middleware design that still pose a high entry cost for RFID technology adopters [4].

The Second-Generation RFID (2G-RFID) [5] systems contain not only static information such as object identification and description, but also introduce dynamic rules in encoding tags reflecting the up-to-date service requirements [6] and dynamic network RFID sensor configuration. In practice, an RFID system always works in an environment with multiple reader sensors and multiple protocols [7]. In order to ensure the system can adapt to all working environments, RFID middleware should be used because it provides the applications with a device-neutral interface to communicate with different hardware [8]. The middleware design must be full-featured, fully compliant with international standards, and able to support simultaneous communication of multiple applications with multiple types of RFID hardware. EPCglobal standards, which are globally accepted standards that ensure global applicability, have been widely adopted by RFID middleware products. EPCglobal Inc. leads in the development of industry-driven standards for Electronic Product Codes (EPC) to support RFID. It is driving the global adoption of EPC as a global standard to enhance visibility of products. However, some shortcomings can still be found in the standards [9]. For example, they are complex, bulky, expensive, and they are lacking in advanced system management features. It seems that the standards are not suitable for heterogeneous uses and for small and medium-sized applications. Simplicity and flexibility, fulfillment of specific needs, and easy implementation should be included in the design of middleware for small-sized and medium-sized applications [10].

To be able to manage these issues we have developed the Data EPC Acquisition System (DEPCAS). It represents an approach to solve several of the open issues and to adapt requirements for the new RFID system generation. The DEPCAS approach involves a novel software architecture to solve RFID middleware which is based on the modern Supervisory, Control and Data Acquisition (SCADA) software architecture. The basic concepts of DEPCAS are described in [11], which includes a comparative analysis of the DEPCAS and SCADA architectures that proves the adequacy of adapting a well known and classical solution for conventional industrial applications to solve the recent challenges arising in RFID environments. Nevertheless, there is a lack of implementation details in this previous article that can be considered as a starting point of the work presented here. In this sense, this work is devoted to describing the design and development details of an important part of the DEPCAS middleware: the so-called RFID Sensors Networks Management (RSNM), which is especially concerned with how generic RFID sensors (edge readers, readers, PLCs…) are organized and managed. This function is included in DEPCAS design as a generic layer called MDM (Middleware Device Manager) that solves two main questions: hiding hardware RFID sensor deployment and hiding communication between RFID sensor reader devices and the RFID middleware In short, the proposed solution and developed in this article is built on top of two key and new concepts:

- Minimum Access Reader Commands (MARC), which is the independent device layer that allows automatic connection and discovery of RFID reader sensors inside an acquisition network.
- RFID Reader Topology Language (RRTL), which is the language used to define and relate RFID sensor networks inside the acquisition middleware.

MARC and RRTL are the main elements in the Middleware Device Manager in DEPCAS support multi-protocols and all data processing tasks, such as filtering, grouping and duplication removal. They must guarantee that a variety of data can be read by the multi-readers and the received data can be extracted, decrypted, filtered and converted to generate the input for the Middleware Logic Manager: the consolidated WHO-WHEN-WHERE.

The rest of the paper is organized as follows: we introduce related works concerning existing RFID middleware solutions and their comparison with DEPCAS in Section 2. Section 3 describes the main issues of our approach to generic RFID reader sensor management inside DEPCAS. Sections 4 and 5 are devoted to presenting the specifications and implementation details of the MARC and RRTL solutions, respectively. Finally, Section 6 exposes the principal conclusions of this work as well as some open questions and future work related to them.

## 2. RFID Middleware Review

The need for RFID middleware is gradually becoming a basic question for non-trivial RFID installations [12]. This is particularly true in heterogeneous environments, which can include multiple readers, application instances, complex processes and sophisticated business semantics. RFID middleware is indispensable for the following main reasons:

1. The need for filtering RFID data in order to avoid redundant or erroneous information that is not required in business applications, while at the same time allowing optimization of resources.
2. The need for processing and interfacing original RFID data (tag reads) in heterogeneous deployments (multi-tagging and multi-readers systems) without resorting to business integration logic.
3. The flexibility of integrating RFID system to support auto-identification in different applications and process models.

### 2.1. RFID Middleware Taxonomies

There are several surveys in the literature [13–17] that propose system taxonomies and compilations concerning RFID middleware. The RFID middleware market is dominated by three tendencies:

#### 2.1.1. Specialized Companies Which Offer RFID Middleware

These companies can be classified as product oriented or specific RFID middleware producers. If they are reader/antenna, or general hardware producers, then they typically include specific RFID middleware to support the hardware. If we speak about a general middleware producer it is quite common for them to have included in their product one specific plugin to acquire RFID data. There are also new companies invoved with the specific commercial target of creating, distributing and installing RFID middleware. We can mention in this group:

- WinRFID^®^ from the UCLA-WINMEC Research Lab. WinRFID is a research result to solve RFID features like scalability and administration, event and data intelligent processing and dispatching. WindRFID proposes a middleware stack approach, with RFID hardware management in the lower layer including directed API calls from reader vendors.
- The OAT Foundation Suite from OAT, a division of Chekpoint, is an RFID middleware platform that includes two-way process integration between enterprise applications and RFID acquisition systems. This middleware includes an OATdevice manager to integrate heterogeneity and OATenterprise to solve the problem of distributed RFID acquisition based on Auto-ID deployments.
- DETEGO^®^ You-R OPEN is the RFID middleware product from RF-iT Solutions based on three main functions: business integration, data management and device management. The device management includes a direct API call from reader vendors and a business integration of the topological middleware organization to distribute information.

#### 2.1.2. Giant Software Vendors Integrating RFID Middleware

There are several examples in this section such as IBM, Microsoft, ORACLE/Sun Microsystems, or SAP. During the last decade each one of these huge companies has dedicated important resources to include RFID acquisition in their own products. Among these RFID middleware products we can mention:

- RFID and Sensor-Based Services from ORACLE/Sun Microsystems. With the fusion of ORACLE and Sun Microsystems, the RFID middleware from both companies was renamed. Now the product name is ORACLE Sensor Edge Server based on ORACLE Fusion Middleware. This version includes *ad-hoc* drivers for the set of specific RFID sensors integrated in the acquisition network and the data distribution is provided by the ORACLE Fusion Middleware and Oracle Enterprise Manager.
- SAP Solutions for Auto-ID and Item serialization is a set of components to include RFID technologies in SAP management tools. The solution offers three major components: SAP Auto-ID infrastructure, SAP object event repository and SAP event manager. The SAP Auto-ID infrastructure provides solution packages including predictive maintenance, field service management and adaptive configuration. The complete suite is Auto-ID focused, integrating the EPC Global network for data distribution.
- IBM WebSphere RFID is the IBM middleware to implement RFID acquisition. It consists of EdgeControllers and the WebSphere RFID Premises Servers which includes WebSphere Application Server and DB2 Workgroup server. The IBM WebSphere is included in InfoSphere Traceability Server that supports device management and data capture applications, without topological configuration.
- RFID Anywhere from Sybase iAnywhere is a software infrastructure that simplifies the development, deployment, configuration and management task for RFID networks. This software infrastructure has been integrated into the SAP Auto-ID solution.

#### 2.1.3. OpenSource RFID Middleware

These proposals are research results that originated in university groups or consortiums. There are several Open Source initiatives aiming to provide RFID middleware:

- CUHK RFID Middleware from the MobiTEC Technologies Centre of the Chinese University of Hong Kong [18]. The CUHK RFID integrates a restricted ALE implementation and specific CUHK reader compliant with ISO18000-7 protocol.
- ASPIRE RFID Middleware [19] is a EU funded project to deliver an Advanced Sensors and lightweight Programmable middleware for Innovative RFID Enterprise application as an open source middleware. The ASPIRE project includes a hardware abstraction layer based on Fosstrack libraries to implement the EPCglobal Reader Protocol and EPCglobal Low Level Protocol.
- RIFIDI Edge Server [20] is a software architecture proposed by the RFID Research Center at the University of Arkansas. This middleware defines a Sensor Abstraction Layer to call the reader API, following specific reader protocols, the EPCGlobal Low Level Protocol or any other *ad-hoc* reader acquisition driver. There is no support for topological network configuration.
- FOSSTRAK [21] (previously ACCADA) is a middleware implementation sponsored by Auto-ID Labs oriented to produce free and open source software for track and trace. It includes a Low Level Reader Protocol Commander that provides a LTK-XML editor, a GUI LLRP packet editor and a reader navigation console.
- DEPCAS [11] is a general purpose middleware proposal inspired by the modern SCADA software architecture. This software architecture is well known for offering successful solutions for conventional industrial applications.

#### 2.2. RFID Sensors Network Management

The study and comparison of the previous RFID middleware in relation with the RFID sensors network management allows obtaining a set of features that are typically included in this kind of middleware systems. Among these features we include:

- *Device Agnostic*: the device management should include a specific function to hide the alternatives of existing devices related to physical parameterization, communication protocols, communication options, *etc*.
- *Configuration and parameterization of RFID devices*: an agnostic approach to existing devices sets the options to manage from a homogeneous point of view the configuration and parameterization of every piece of equipment in the RFID acquisition network.
- *Topological RFID devices*: the RFID device management can include some network organization that provides additional capabilities to the acquisition network such as redundancy, input/output semantic function, parallel reading devices, *etc*.
- *Start-up devices*: the set of operations to start-up and configure and RFID sensor inside a network acquisition are known as starting up protocols. Some of the existing RFID middleware support a starting up protocol by giving instructions about how to include RFID sensor in the network.
- *Report RFID sensors data*: the deployment of RFID sensors required to introduce maintenance operations in the network. Statistics and reporting information are essential to optimize these operations.
- *Monitoring devices in live operation*: some existing RFID sensors allow supervisory management from middleware while the device is working.
- *Diagnosis of devices in live operation*: the RFID middleware includes operations like device signaling, log warnings, updating software sensors, *etc*.
- *LLRP implementation*: the Low Level Reader Protocol is the standard proposal from EPC Global Network to specify the interface between RFID readers and any client software.
- *OSGi Technology*: the increase of software complexity in the heterogeneous hardware environment has promoted the development of frameworks to support continuous changes. The OSGi framework is a module and service platform that implements a complete and dynamic model. Any sensor that implements OSGi provides an environment for modularization of applications in small pieces tightly-coupled, dynamic loadable and configuration files to declare external dependencies. The OSGi Architecture is one of the most common specification frameworks used in our days. The OSGi specification enables components to hide their implementation from other components while communicating through services.

### 2.3. DEPCAS General Structure

**Figure 1.**
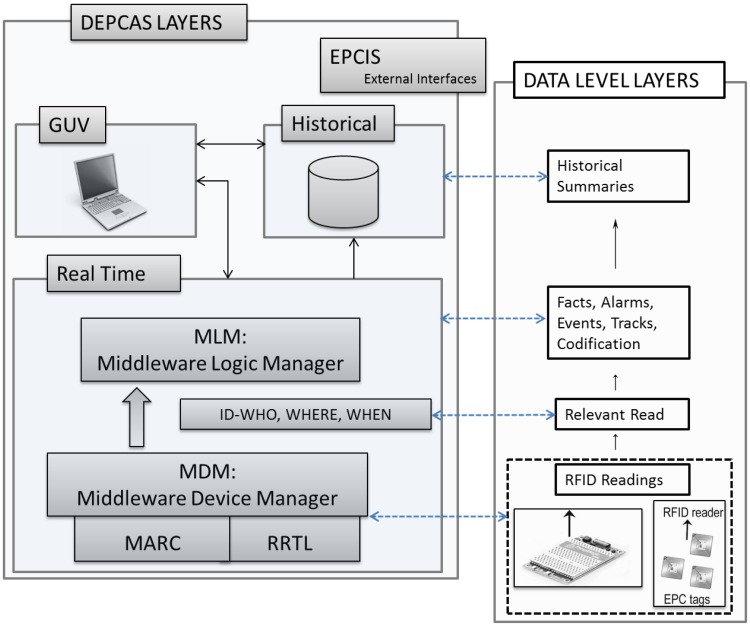
DEPCAS Architecture.

A brief summary of the highlights of DEPCAS is presented in this subsection. The purpose is to show the general block organization of this middleware solution in order to locate the specific part of it which is the main subject of this work. In any case, a major overview of DEPCAS can be found in [7]. DEPCAS is a middleware designed to solve RFID acquisition issues in heterogeneous and real systems, as described in Figure 1. The scheme shown here is based on the architecture of modern SCADA systems.

The main features in DEPCAS are:

- Producing RFID processed data.
- Hiding heterogeneous RFID deployment systems with a homogeneous layer approach.
- Translating business (async or sync) needs to RFID systems.
- Providing management capabilities in RFID middleware environment.

To solve these main targets the structure of the acquisition system is organized in four major layers or subsystems:

1. Middleware Device Manager (MDM).
2. Middleware Logic Manager (MLM).
3. Graphical User Viewer (GUV).
4. Information system exchange (EPCIS: EPC Information Services).

MDM has the following basic functions: first, the communication management with one or more devices (RFID readers). Second, the implementation of the communication protocol with the readers and the event processor implementation (Application Level Event or ALE used in the EPCglobal standard), and third, support for topological configurations defined by the acquisition equipment: antennas, edge readers and readers.

MLM supports the process to obtain RFID summarized data from auto-identification raw acquired data. The logical process to be resolved will depend on each particular scenario. This process will generate information of a permanent nature which is the result of the process in every scenario instance. The basic scenarios considered are related to monitoring and tracking operations, aggregation of information items to generate new information, sorting of items, raw format RFID data conversion, and machine status delivery information. The concept of scenario is equivalent to a logical process that generally can be applied in all situations where information is used for self-identification. At each stage information is received, processed, summarized and accumulated, and finally the data is generated and consolidated according to a specific logic.

GUV is the mechanism that DEPCAS foresees to help install and manage an RFID system from the engineering point of view. This subsystem supports two basic operations: the supervisory capability of the RFID system and certain data manageability features (configuration and acquisition data).

EPCIS services should be the gateway of information between the data provided by the MDM and different MLMs to external business. These services will enable both receiving and sending information from the DEPCAS system to other systems. Table 1 compares the features supported by DEPCAS MDM to some of the previous RFID proposals:

## 3. RFID Sensors Networks Management Specification

As it can be seen from the previous introduction, MDM, the first layer of DEPCAS architecture, copes with the management of the RFID information generated at the lower level. RFID data acquisition and initial processing must be carried out at this stage, which is directly related to the management of RFID sensor infrastructure used in a given application. For that reason a more precise version of this DEPCAS architecture module is called RFID Sensors Networks Management (RSNM). This seems to be a more appropriate notation when multiple RFID readers from different vendors located at different spatial positions must be taken into account in a specific application.

Nevertheless, a more detailed specification of the RSNM is required prior to any kind of implementation. This Section is devoted to formalize our specification proposal. A specification like that can be solved depending on the most varied aims: architecture, network dedication, logical network organization, specific network or application requirements, size, *etc*. To annotate the problem we suggest some definitions that restrict the network specification that allow handling our RSNM proposal in a better way.

### 3.1. Definitions

Definition 1: RFID Sensors Network (RSN). An RFID Sensors Network (RSN) is defined by the pair of sets: RSN = {R, T}, where R is the set of RFID sensors to manage inside the network and T is the set of RFID tags covered in a specific timestamp (t_i_).

#### 3.1.1. Operations to Manage T inside R

In order to classify the operations defined from the sensors set to the tags inside an RSN we define three kinds of devices: input sensors (R_i_), output sensors (R_o_) and neutral sensors (R_n_). The elements of R can be defined in terms of his network function as:

(1) $$\text{R}=R_{i}\cup R_{o}\cup R_{n}$$

An RFID reader belongs to the input sensors set if it introduces tags inside the tag set (T) of network covered tags. An RFID reader belongs to the output sensors set if it deletes tags from the tag set of network covered tags. If a reader does not perform any action in the tag set then it is included in the neutral sensors set. The tag addition or subtraction operations are defined as atomic operations. If a tag is yet included in T then it is not included. If a tag is not yet included in T then it is not deleted.

#### 3.1.2. Operations to Manage (*r,t*)∈(R×T)

In relation with how to manage the read inside the RSN we define two kinds of sets: redundancy sets and filter sets. By redundancy set we refer to a subset of the product of R × T where the result allows expressing a set of reading redundancy. Formally, a redundancy set is defined as:

(2) $$\forall (\text{r},\text{t})\in ({\text{R}}^{\prime }\times {\text{T}}^{\prime })[r\in R^{\prime }\wedge t\in T^{\prime }],{\text{R}}^{\prime }\subset \text{R},{\text{T}}^{\prime }\subset \text{T}\wedge {\text{R}}^{\prime },{\text{T}}^{\prime }\neq \emptyset$$

where one tag (t) that is read inside the product is consolidated in the acquisition system only if it is read by all the readers from R in the redundancy set.

We express a tag filter as a product of R × T where the result allows defining a reading filter of a tag inside a set of sensors. Formally, a filter set is defined as:

(3) $$\begin{array}{cc} \forall (\text{r},\text{t})\in ({\text{R}}^{\prime }\times {\text{T}}^{\prime })[r\in R^{\prime }\wedge t\in T^{\prime }],|{\text{T}}^{\prime }|=1, & {\text{R}}^{\prime }\subset \text{R},{\text{T}}^{\prime }\subset \text{T}\wedge \text{R} \end{array}$$

where the tag filter is defines in term of R′⊂R, T′⊂T and R′ T′≠∅, and the cardinality of T′ is 1, where if the tag defined in the filter is read inside the product R′ × T′ then is consolidated inside the acquisition system, in case of the tag is not defined inside the product then it is ignored.

#### 3.1.3. RSN Polling Options

Related with the way a reader inside an RSN acquires the tag reads we define two reading philosophies: polled scheme and exception scheme. A reader is said to use the polled communication scheme where the reader is in total control of the communication systems and makes regular (repetitive) requests for data reads. The set of tags under coverage is defined by the hardware according to their own configuration (interval time under coverage, exception, the number of reads inside the coverage, under coverage from different antennas, *etc.*), but it is only read according to the polled scheme defined for the reader.

A reader uses the exception scheme, or unpolled communication, if every tag read (with the same idea as before) under coverage generates a tag read. This technique reduces the unnecessary transfer of data, but it demands that a reader exception policy be defined to consolidate data.

The DEPCAS Middleware Device Manager (MDM) can be defined as a set of one or more RSN that acquire RFID tags and create n-tuples of who-where-when (tag-RSN-timestamp) that are processed by the middleware logic manager MLM to generate business related data. The consolidated data is defined by a WHO (tag), a WHERE (a logical reader position) and a WHEN (timestamp). This record, called relevant record (RR), is the result of filtering and smoothing physical reads, solving false reads, repetitive reads, hardware specific characteristics or any other inconsistencies that topological definition can solve.

In order to manage RSN we can define three types of RSN relations: concentrator, master/submaster and peer to peer. These types of RSN allow defining acquisition and distribution systems in extended RFID installations.

Definition 2: RSN Concentrator. An RSN is a network concentrator if it receives data from different RSN to consolidate data and generate RR. In terms of RSN definition a system is formally RSN-Concentrator if RSN_C_ = {RSN_1_, RSN_2_, …, RSN_m_} where RSN_i_ = {R_i_, T} do not consolidate information, and every RSN_i_ manages the same tag set, T.

Definition 3: RSN Master/Submaster. A master/submaster RSN is a RSN that receives consolidate data from *n* RSN. A “master” RSN system defines a set of readers that are inside others RSN (not all, may be just a subset from RSN_i_) and a set of tag included in other RSN_i_. The master/submaster RSN systems supports hierarchic organization when there are local RSN associated to local installation (submaster) and some of the acquired information needs to be forwarded to a central (master) RSN system.

Definition 4: RSN Peer to Peer. The peer to peer RSN system allows to share data from two completes RSN. Two systems RSN_1_ = {R_1_, T_1_} and RSN_2_ = {R_2_, T_2_} work as peer to peer if there are a reader set R′ and a tag set T′ such that R′ is a common subset from R_1_ and R_2_ and T′ is a common subset from T_1_ and T_2_. The peer to peer configuration allows sharing data between departments (intra-organization) or organizations (inter-organization) solving only the acquisition process and letting the business process to the specific middleware environment.

### 3.2. RSN Topologies

The basic proposal in DEPCAS-MDM includes the following RSN topologies:

- Point to point.
- Multipoint.
- Relay connection.
- Store and forward relay operation.
- Talk though repeaters.

The point to point is the simplest configuration, where data is exchanged between middleware and reader stations. The acquisition middleware can be set up as the master, and the RFID readers as the slaves. It is possible for the middleware to communicate in full duplex mode (transmitting and receiving) with all the readers.

The multipoint is the network topology where data is exchanged between middleware and reader stations using a shared communication channel. This is a more complex arrangement requiring sophisticated protocols to handle collisions between two different readers wanting to transmit at the same time (normally using TCP/IP stacks).

The relay connection topology uses RFID middleware to retransmit RFID acquired data to other RFID middlewares. There are two possibilities here: store and forward relay operation and talk through repeaters. The store and forward relay operation can be a component of the other approaches discussed above, where one RFID reader retransmits messages to another RFID network. In these situations RFID readers work as “edges readers” and they are not connected with the middleware; there are other readers that concentrate all RFID data. The other option allows extending RFID networks using RFID switches that retransmit the reads from a network to another.

## 4. MARC: Minimum Access Reader Commands

MARC is the minimum set of instructions that an RFID reader must supply to support the operational instructions inside a network. A previous analysis of how real RFID readers are used in real implementations must be considered before addressing what such minimum set must be. In fact, what we propose is to find a response to the question of how heterogeneous RFID reader devices can be managed in a uniform way.

### 4.1. Using RFID Readers

Real RFID middleware implementations mix sets of different equipment. Employing RFID readers from several vendors can impose significant complexity to a system. To solve this problem there are two possible solutions. First, define a specification that all vendors should implement so that there can be a single way of interacting with such a device. Such specifications exist *de facto* for RFID UHF readers, namely the EPCglobal Reader Protocol (RP) and EPCglobal Low Lever Reader Protocol (LLRP). There are two main questions about these specifications. First, not all vendors implement them. Second, LLRP and RP are extended specifications that trend to cover many theoretical situations. In these cases we have to define an abstraction layer, hardware abstraction layer, HAL, that can translate proprietary vendor system calls to standardized calls (e.g., to RP calls).

An RFID reader reads passive tags in the field of view of one or more antennas. The main steps required to achieve this result are:

1. Set up the reader by setting initial parameters (configuration).
2. Start an inventory scanning operation at the appropriate moment.
3. Select an antenna (if the reader can connect to more than one).
4. Send one or more commands to cause tags to reply with their ID's.
5. Transmit a continuous-wave signal while listening to tag responses.

Optionally, once a specific tag has been identified, perform additional operations on that tag, such as memory reads or writes, or changes in the tag configuration to consolidate the information.

Report the results of this register attempt to a host or controller: what tags were read, what other operations were performed, and the results thereof.

The configuration or setup operation loads the basic parameters in the readers from the reader master station. Among these parameters we can mention frequency (if the reader is multi-frequency), communication profile (serial, TCP/IP, …), antenna configuration, working time-outs, …

The antenna reader use radio signals as waves; these waves can circulate long distances and affect the operation of adjoining readers (and any other devices operating in the same radio band). The antennas commonly employed are not directional, and the radiated waves can rebound from objects and people, so that tags on items outside the normal read zone will sometimes be detected. A reader should not be on unless there is something to read in the intended location. In many cases, a simple sensor (e.g., a motion sensor or photocell) may be employed to signal when it is likely that tags are in position to be read; this sensor can then be used to trigger the reader. The reader might also be triggered at known times if the operation being monitored is sufficiently regular to allow it. Alternatively, the controller may take care of deciding when reading should take place, and simply expect the reader to act as soon as it receives the appropriate command from the controller.

Passive tags depend on power received from the reader to operate their integrated circuits. Before a population of tags is ready to respond to commands the reader must first transmit a non-modulated signal for some period of time. The duration of this power period is usually specified by the protocol and needn't be configured by the controller. However, there is a general set of options for the data rates and modulation to be used by the reader and tag:

- Reader data rate: The duration of a binary “0” character. Faster data rates allow us to count more tags per second, but increase bit errors and decrease read range.
- Tag modulation rate: The rate at which the tag changes the scattering state of its antenna.
- Tag coding: tags also have several options for how to encode data sent to the reader.

The tag and reader modulation and timing choices are communicated by the reader to the tags during the initial inventory commands. The optimal choice of parameters varies depending on the application, so the controller must be able to configure these parameters before an inventory operation is launched. Even after the population has been limited by Select commands, there may be a large number of tags that can respond to a query from the reader. Protocols must make provisions to resolve potential collisions between tags sharing the same wireless medium.

Once a tag is successful and replies without colliding with another tag, the reader generally requests the tag's identifier. The tag has been consolidated. At this point, the reader can optionally ask the tag to provide a random number, the handle, which it uses to communicate with that specific tag. The reader can read any part of the tag's memory that is not locked against reading, write to any part of the tag's memory that is not locked against writing, change lock status of memory segments if it has the needed passwords and the status has not been previously fixed. Access operations must essentially be performed in real time, just after a tag of interest has been consolidated. Thus an instruction to access a tag must essentially be pre-existing, triggered immediately upon consolidating a suitable tag.

Collecting tag data is of little benefit if it is not communicated from the reader to where it can be used. Readers must have a scheme for reporting what they've found. The contents of the report could simply be a list of each valid EPC read during a specific sequence of inventory operations, or it could be much more elaborate. If we are trying to debug the operation of the reader, we might also want to know how many inventory attempts were made, how many times each tag was read and what value of Q was used in each attempt. We might also want to know how many slots contained tag replies, no replies, collisions between tags, and so on. If we are trying to guess at the physical location of a tag, we might also want some measure of the signal strength of the tag's response. The controller needs to be able to select the contents of the report in order to provide information the user needs without wasting time on unneeded data.

### 4.2. MARC List

A set of common instructions can be extracted from the previous analysis. The specific list of instructions that we propose allows hiding heterogeneous devices inside a homogeneous framework. This MARC list is closed and gives an independent layer of RFID management. The implementation of MARC is related to RFID reader API and gives a dependent hardware layer. The basic configuration commands included in MARC and their respective explanation are the following:

- *setReaderId*: set identification data for a specific reader.
- *setLineType*: set data line type. We manage three communication line types: poll line for standard communication, dialup line for temporary line connection and listen line for lines where the RFID host server interprets data on the communication line but does not send a poll request or acknowledge that it received polled data.
- *setConnectionType*: set the connection type for an RFID device. There are two options: serial connection or network connection.
- *setUsername*: identify the user reader access.
- *setPassword*: identify the password reader access.
- *setNotifyFormat*: configure the format message to be used by the reader.
- *setNotifyTime*: establish the period that the reader notifier should use.
- *setPollConfiguration*: set the specific poll configuration including: baud rate, communication retries, extra Tx/Rx time, delay between readers I/O operations, RFID reader address, time-out no-reply RFID reader, RFID reader poll and offline delay, poll schedule RFID reader, …

In order to change the way an RFID reader is working we can use the following MARC commands:

- *setAntennaSequence*: set an order sequence to read from multiple antennas installed in one reader.
- *setAutoFalsePause*: order to autonomous function mode to pause working.
- *setAutoMode*: start/stop autonomous reader functions.
- *setAutoStartTrigger*: activate the trigger sending functions in reader autonomous function.
- *setAutoStartTimer*: activate periodic timer reader function in reader autonomous function.
- *setAutoStopTimer*: reset periodic timer reader function in reader autonomous function.
- *setAutoStopTrigger*: reset the trigger sending functions in reader autonomous function.
- *setAutoTrueOutput*: order to autonomous function mode to process tag reads.
- *setAutoTruePause*: order to autonomous function mode to start reading process.
- *setConnect*: establish a communication connection.
- *setNotifyAddress*: init a listening TCP port to instruct the reader to send notification messages.
- *setRun*: start to execute a configuration reader mode.
- *setStop*: stop to execute a configuration reader mode.
- *setTime*: synchronize reader time.

The implementation of MARC sentences has been solved using pipeline processing [22] where the elements of the pipes are programs (string containing links or MARC sentences) and commands (links to compose sentences or programs). A command comprises a series of process steps to be executed sequentially in the presence of an event. A program or chain consists of one or more commands, which are the links in the chain. Each command runs a simple process of the program processed. The pipeline architecture gives a support structure to systems that process data flows. This is the case of the Pipe-DEPCAS system, which flow of information between the RFID tag readers and the specific purpose processing systems. Every process step is encapsulated in a so called filter component. The data is transferred across pipes between adjacent filters. The recombination of filters allows constructing families of related filters. Every step is processed the same way according program instructions and the configuration of different RFID readeR′s characteristics are defined throw an XSD scheme. An example of scheme configuration for an ALIEN-9780 reader integrated in DEPCAS process is presented in Table 2.

## 5. RRTL: RFID Reader Topology Language

### 5.1. Description

RRTL is a language to define RSN topologies. The basic idea included in a RRTL is to define every element included inside an RSN. The RRTL has sentences to define the elements in the network, the relations between these network elements, and the basic actions to do with the network tags to consolidate RFID data.

Generally a Service Oriented Architecture (SOA) provides interoperability by defining interfaces and protocols independently. In the Web Service Resource Framework (WSRF) the semantics of a resource—its operations and arguments—is described by using a Resource Property XML Schema inside a WSDL document. By using RRTL to describe the semantics and behaviour of a resource, specifically a sensor network paradigm service, it is possible to create “dynamic” services that react to each input in a different way.

In the basic RRTL structure we have included an approach, seen as a semantic language standard, that aims to serve the RSN domain specifically by providing schemata to manage the interaction between an RSN and an RFID middleware provider. By using these schemata it is possible to describe the service capabilities, semantics, functions and parameters in a client interpretable way. This approach is called Dynamic Service Evolution (DSE) [23] because a resource can change its semantics dynamically with respect to a defined schema. In such a case no adaptations are needed on the client side. The whole DSE process is illustrated with the following steps from the client and the service point of view.

- The client contacts a Web Service found via a broker or registry to obtain semantic, self-describing information of that service. This information is called a description and specifies a service beyond its pure interface description.
- The Web Service returns its semantics represented in a client interpretable format, namely a Dynamic Service Description (DSD). This response can either be processed automatically or by using a GUI representation that allows for user interactions.
- Based on the user input or the automated processing the client produces a definition document and a data document that are both sent back to the service. We name this combination a Dynamic Service Interface (DSI).
- In the last phase the Web Service delivers a service instance and an optional result document that might contain some presentable data in form of two dimensional diagrams, tables, …

The supported dynamism is much more powerful than the usage of ordinary service data and interfaces only, because of the following reasons:

- By decoupling the parts of one language into different schemata only a smaller part of the system has to be changed or extended in the case of a schema change.
- The client can implement and interpret different semantic schemata (descriptions) and map them to one common service interface.

Before starting with a detailed discussion, included in the next section, a few factors that should be borne in mind when designing the system are:

- Simplicity (the ‘KISS’ principle: Keep It Short and Simple).
- Minimum response time.
- Deterministic type operation.
- Minimum cost.
- Optimum efficiency of operations.

While it is difficult to generalize all RFID readers and communication systems forming part of an RSN system, a few considerations are discussed below:

- Station address. Every reader (and edge reader) must have a unique address.
- Protocol message retries. How many retries or messages transmitted before the reader station flags as unavailable?
- Timeout delay. The timeout delay for a message received from an RFID reader.
- Size of messages from RFID reader. This defines the maximum size of messages allowed from the reader during a poll by the master station.
- Priority message transmit. This defines when an immediate message is required to be transmitted by the master station, at the conclusion of a poll of a reader station or when the master station appears in the poll sequence.
- Poll sequence. Define the reader addresses in the poll sequence for both priority and normal message transfers.

### 5.2. RRTL Elements

**Figure 2.**
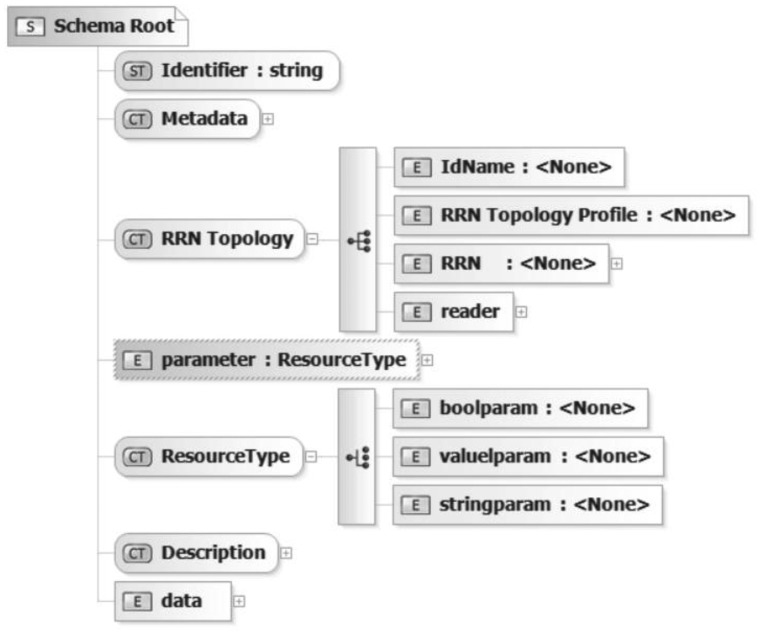
RRTL main schema.

The description schema shown in Figure 2 builds the first part in the row of schemata that required supporting dynamism of a RRTL specification creation.

**Figure 3.**
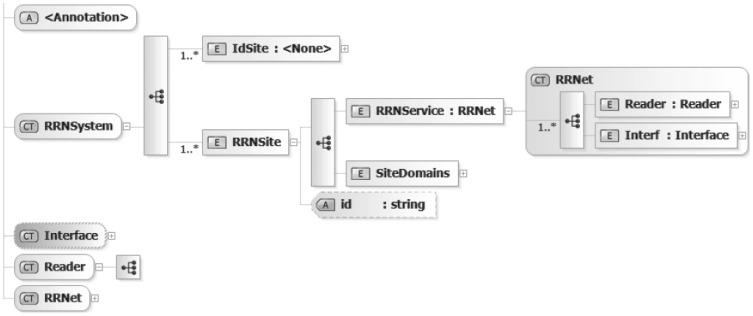
RSN topology schema.

The identifier field is used to identify an RSN implementation. The metadata section element encapsulates some other field to describe the service implementation and its mode of operation. The RRN topology field gives the user a hint about the realized RSN type while the name extends this information and specifies the service in a more precise way. However with the usage of the description field it is possible to describe the application area and domain of the RSN solution. The parameter section holds elements of type valueparam, boolparam or comboparam. The first one allows specifying floating point numbers, the second one logical value selection and the third one represents a list of items. The data block specifies the records that should be provided for the specific network elements. There are several complex types managed inside RRTL schemata. At this point we present the most relevant is the RFID Reader Network (RSN) illustrated in Figure 3.

The RFID Reader Network is defined as a complete system inside the DEPCAS middleware. The system can manage a set of RSN sites. Three types of information are required to completely define the relationships within A RSN system. Each type corresponds to a successively larger zone of interest. The information distributed at each level becomes more comprehensive, with each level building upon the information gathered from the previous level. The three types, in ascending order of complexity, are: RSN service is a single RFID network service. The RSN site consists of a group of RSN services. The site may be capable of full RSN functionality, or it may be used for non-operational purposes such as training, test or simulation support. The various services which comprise a site are usually located in one physical network location. In contrast, different sites are usually located in different places. The RSN system consists of a primary site, plus optionally other different functional sites: backup site, redundant site or satellite site.

## 6. Conclusions

RFID middleware research is oriented to find new alternatives to solve the best way to include auto identification in productive processes. The new topological readers network and the heterogeneous business application must be connected though RFID middleware solving all the established requirements. In this paper, regarding the development of MDM for RFID, there are two main focuses. One is to build an agnostic RFID device acquisition system that at the same time meets the existing standard proposals. The other one is how to manage topological RFID network questions like RFID levels of reader points (antennas, edge readers, reader, PLC with RFID readers, *etc.*) or redundant reader points. These two main subjects are covered in our solution DEPCAS MDM: one with the use of MARC to hide device management or standardization protocols, and other for topological questions with RRTL to express network configuration.

Concerning future works there are different issues to be resolved. First, we want to extend the MARC functionality to new devices and new technologies. We also want to extend the RRTL functionality to new topological structures (RFID matrix networks, flow oriented networks, RFID integration in workflow processes). Second, there is one important issue to be solved that is the adaptability and the flexibility in the context: detect and automate the RFID topological installation inside a RFID reader network.

## Figures and Tables

**Table 1.** DEPCAS MDM support comparison to related work.

	**FOSTRACK**	**RIFIDI**	**ASPIRE**	**ORACLE**	**IBM**	**DETEGO**	**OAT RFID Plat.**	**DEPCAS**
Device Agnostic	Yes/no	No	Yes	Yes	Yes	Yes	Yes	Yes ^1^
Device Configuration	Yes	Yes	Yes	No	No	Yes	Yes	Yes
Topological Configuration	No	No	Yes	Yes	No	Yes	Yes	Yes ^2^
Need Programming Starting-up device	Yes	Yes	Yes	No	No	No	No	Yes/No ^3^
Statistical Data from device	Yes	No	Yes	No	No	Yes	Yes	Yes ^4^
Monitoring Data from device	No	No	Yes	Yes	Yes	Yes	Yes	Yes ^5^
Diagnosis capabilities	Yes	No	No	No	Yes	No	Yes	Yes ^6^
LLRP implementation	Yes	Yes	No	No	No	No	No	No
OSGi Technologies	No	Yes	No	No	No	No	No	Yes ^7^

1 Using MARC sentences to configure and connect.

2 Using RRTL.

3 Depending on the API of the chosen reader.

4 Statistical operations are included in MARC sentences.

5 MARC sentences include data quality evaluation if it is possible to obtain information from readers.

6 There are MARC sentences producing device diagnosis and there are operations included in RRTL about topological diagnosis.

7 To process parallel RFID readers.

**Table 2.** MARC schema configuration of ALIEN RFID 9780.

<reader driver-class=“mdmALIEN9780Setup”
type-class= “mdmALIEN9780Setup” name= “ALIEN9780_0”
organization= “UNED”
campus= “28040-Madrid/Univ.Complutense”
address= “c/Juan del Rosal 16”
dept= “ISSI”
location= “Planta: 1, Desp: 112”
use= “Operativo”
auto-mode= “true”
auto-interval= “1000”
auto-chain= “Test” >
<arg>62.204.207.74</arg>
<arg>23</arg>
<arg>alien</arg>
<arg>password</arg>
<property key= “setNotifyAddress” value= “localhost:9001” trim= “false”/>
<property key= “setTime” value= “2012/1/31 00:00:00” trim= “false” >
<property key= “setAutoStartTrigger” value= “0,0” trim= “false”/>
<property key= “setAutoTrueOutput” value= “1” trim= “false”/>
<property key= “setAutoTruePause” value= “500” trim= “false”/>
<property key= “setAutoStopTimer” value= “1000” trim= “false”/>
<property key= “setAntennaSequence” value= “1,0,0,0” trim= “false”/>
<property key= “setAutoMode” value= “1” trim= “false”/>
<property key= “setPollConfiguration” value= “1000” trim= “false” >
<property val= “setConnectTCP” value= “62.204.207.74:23” trim= “false”/>
<property val= “setUsername” value= “alien” trim= “false”/>
<property val= “setAntennaLocation-00” value= “UNED-ISSI Dept. main entrance door” trim=”false“/>
<property val= “setAntennaLocation-01” value= “UNED-ISSI Dept. boss room door” trim= “false”/>
<property val= “setAntennaLocation-02” value= “UNED-ISSI Library main entrance door” trim= “false”/>
</property> val= “setAntennaLocation-03” value= “UNED-ISSI Library exit door” trim= “false”/>
</property>
<property key= “setPassword” value= “password” trim= “false”/>
<property key= “setRun” value= “false” trim= “false”/>
</reader>
